# BSXplorer: analytical framework for exploratory analysis of BS-seq data

**DOI:** 10.1186/s12859-024-05722-9

**Published:** 2024-03-04

**Authors:** Konstantin Yuditskiy, Igor Bezdvornykh, Anastasiya Kazantseva, Alexander Kanapin, Anastasia Samsonova

**Affiliations:** 1 Institute of Translational Biomedicine, Saint Petersburg State University, Saint Petersburg, Russia 199004; 2 Laboratory of Neurocognitive Genomics, Department of Genetics and Fundamental Medicine, Ufa University of Science and Technology, Ufa, Russia 450076

**Keywords:** Epigenomics, DNA methylation, Bisulfite sequencing, Exploratory data analysis and visualization, Methylation density, Gene body methylation, Clustering, Gene modules, Non-model organisms

## Abstract

**Background:**

Bisulfite sequencing detects and quantifies DNA methylation patterns, contributing to our understanding of gene expression regulation, genome stability maintenance, conservation of epigenetic mechanisms across divergent taxa, epigenetic inheritance and, eventually, phenotypic variation. Graphical representation of methylation data is crucial in exploring epigenetic regulation on a genome-wide scale in both plants and animals. This is especially relevant for non-model organisms with poorly annotated genomes and/or organisms where genome sequences are not yet assembled on chromosome level. Despite being a technology of choice to profile DNA methylation for many years now there are surprisingly few lightweight and robust standalone tools available for efficient graphical analysis of data in non-model systems. This significantly limits evolutionary studies and agrigenomics research. BSXplorer is a tool specifically developed to fill this gap and assist researchers in explorative data analysis and in visualising and interpreting bisulfite sequencing data more easily.

**Results:**

BSXplorer provides in-depth graphical analysis of sequencing data encompassing (a) profiling of methylation levels in metagenes or in user-defined regions using line plots and heatmaps, generation of summary statistics charts, (b) enabling comparative analyses of methylation patterns across experimental samples, methylation contexts and species, and (c) identification of modules sharing similar methylation signatures at functional genomic elements. The tool processes methylation data quickly and offers API and CLI capabilities, along with the ability to create high-quality figures suitable for publication.

**Conclusions:**

BSXplorer facilitates efficient methylation data mining, contrasting and visualization, making it an easy-to-use package that is highly useful for epigenetic research.

**Supplementary Information:**

The online version contains supplementary material available at 10.1186/s12859-024-05722-9.

## Background

Epigenetic changes, especially DNA methylation, a phenomenon in which a methyl group is attached to a cytosine, are critical for a wide variety of biological processes that are essential for the development and welfare of both plants and animals [1–3]. Cytosine methylation is a common epigenetic mark that is generally found in eukaryotes, including vertebrates, insects, fungi, and plants, the latter exhibiting patterns and pathways of DNA methylation that are distinct from animals and fungi [4–6]. While the core methylation enzymes are ancient and exhibit high levels of conservation, the molecular mechanisms regulating these enzymes to establish DNA methylation patterns that are specific to a cell type or a particular locus can vary significantly. Despite the progress in our understanding of DNA methylation targeting and regulation, many fundamental questions remain unanswered, such as the nature of the signals that instruct DNA methylation, the relationship between methylation changes and neoplastic transformation, and the stability and heritability of environmentally induced DNA methylation. Indeed, DNA methylation plays a crucial role in shaping genome evolution, including transposon silencing [7], gene expression, genome stability [8], and mutation rates. Also, the diversity of DNA methylation across species is shaped by genome sequence evolution. Although model systems have been, and will continue to be, indispensable for fundamental epigenetic research [9, 10], it is essential to consider the importance of non-model species to gain a comprehensive understanding of the regulatory mechanisms involved. With respect to this, comparative studies involving hundreds of non-model species from diverse taxa [9–12], as well as integrative research encompassing tens of subjects from the same species [13, 14] are crucial in enhancing our understanding of the evolution of epigenetic regulatory mechanisms, epigenetic inheritance and, finally, phenotypic variation.

Bisulfite sequencing is a highly robust technology that enables detection and quantification of DNA methylation patterns [15–17]. In recent years, it has made immense contributions to our understanding of gene expression regulation, genome stability maintenance, and the heritability of epigenetic marks [2, 3, 7, 18]. In animals DNA, methylation is predominantly observed at CG sites, while in plants, it is found in three sequence contexts: CG, CHG, and CHH (where H represents A, T, or C). Furthermore, among the three contexts, CG dinucleotide methylation in plants exhibits the highest likelihood of transgenerational inheritance and is thus a prime candidate for epigenetic adaptation.

The analysis of bisulfite sequencing data involves a series of steps, including reads alignment, exploratory data analysis, identification of differentially methylated regions/cytosines (i.e., DMRs/DMCs), functional enrichment analysis, epigenome-wide association studies (EWAS), and data visualization. Over the past decade, numerous computational solutions, of various degrees of sophistication, have been developed for bisulfite data processing and analysis. These solutions include Bismark [19], Bismark-bwt2-e2e, BSMAP [20], BS-Seeker [21, 22] for mapping BS-seq data to genomes, metilene [23], methylKit [24], DSS [25], BSmooth [26] for DMR/DMC discovery, deeptools [27], ViewBS [28], and MethGET [29] for visual exploration of methylation (and expression) data, among others. Additionally, there are various all-in-one solutions for methylation data analysis, including RnBeads 2.0 [30], msPIPE [31], MethylC-analyzer [32], and EpiDiverse Toolkit [33]. While some of these solutions were primarily designed for biomedical applications and can handle methylation data generated with both array and sequencing technologies (e.g., RnBeads 2.0), others (e.g., EpiDiverse [34]) were developed explicitly to conduct EWAS.

These instruments primarily involve the visualization of methylation patterns, identification of DMR/DMC regions, and enrichment analysis, the latter two are usually carried out with the assistance of external software such as metilene and g:Profiler [35]. Such tools are complex, may feature a graphical user interface (GUI), and rely on an array of external packages, libraries and platforms (e.g., Docker). They work very well with datasets generated in model systems, but adapting them to non-model organisms, especially where genomes are not yet available in the UCSC and Ensembl, can be challenging.

In plants, economically important crops, and non-model organisms, visual inspection of methylomes is critical for shaping analysis strategies and selecting appropriate methodologies for data processing. Researchers often rely on custom scripts for methylation profile inspection (see, for instance, here [36, 37], which are rarely published and, thus, are arguably not compliant with the research integrity and reproducibility policies.

Therefore, there is a clear need for a compact, easy to use and flexible tool that can enable researchers to perform exploratory data analyses in both model and non-model systems. It should be able to function independently or be smoothly integrated into epigenomic data processing pipelines.

We have developed BSXplorer to meet this crucial need. This fast and lightweight data mining and visualisation tool is specifically designed to work with BS-seq data and is highly efficient.

### Implementation

BSXplorer is implemented in Pyhton (version 3.9 or higher). The package runs on most modern systems and its functions are available through both Python API and command-line interface. The data processing speed is mostly limited by I/O capacity of the storage. Memory usage is low and for the majority of genomes 8GB of RAM will be sufficient. BSXplorer is publicly available at https://github.com/shitohana/BSXplorer or https://pypi.org/project/bsxplorer/ where a comprehensive user manual is provided. Both source code and test datasets are available at Zenodo repository [38–40] allowing users to download and evaluate the package themselves.

## Results and discussion

### BSXplorer workflow and features

**Fig. 1:**
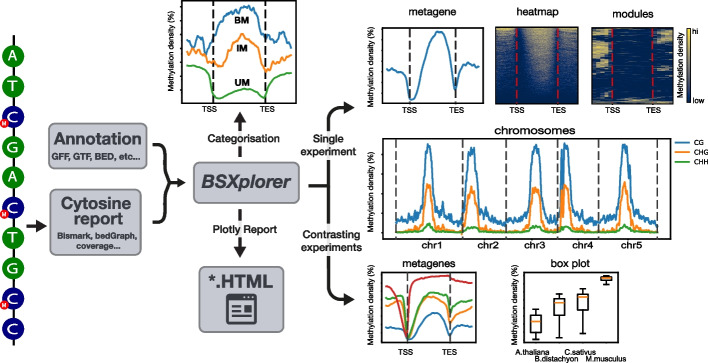
BSXplorer workflow. Aligned bisulfite sequencing data generated by Bismark or other compatible software suites is matched to the annotation of genomic elements/regions of interest stored in GFF/GTF/BED or TSV formats. The BSXplorer tool facilitates the examination of single experiments, as well as the comparison of biological replicates, experiments, and species. The package generates average methylation coverage signal profiles over genomic regions of interest, heatmaps showcasing the overall signal pattern, and can graphically display methylation levels along chromosomes. Additionally, BSXplorer allows for the categorization of regions based on their methylation level and density. The tool supports the export of analysis results and generation of hypertext reports, including interactive versions of profile plots

BSXplorer is a tool to analyse and visualize bisulfite sequencing data from single experiments, as well as for contrasting methylation patterns across different conditions and species. Moreover, this package facilitates the evaluation and categorization of genic and user-defined regions of the genome based on their methylation status with a probabilistic approach [13, 41]. This aids users to gain critical insights into the underlying mechanisms that regulate gene expression. Besides this, the package allows to plot methylation signal across multiple chromosomes. Also, exporting feature enables downstream analyses. The BSXplorer workflow and features are illustrated in Fig. 1.

In order to obtain the aforementioned results, BSXplorer requires processed alignments of bisulfite sequencing data (i.e., whole genome or reduced represented methylation mapping) in a form of either a) a cytosine report, b) bedGraph, CGmap or coverage files, accompanied by a collection of annotated genomic elements in GFF, GTF, BED formats, or a tab-delimited file containing coordinates and IDs of genomic elements or regions of interest. The cytosine report file, a typical output generated by Bismark bisulfite read mapper and methylation caller, contains methylation status for every cytosine in the genome, including strand, trinucleotide context, as well as the coverage counts for methylated and non-methylated nucleotides. The cytosine report can be produced either with *bismark_methylation_extractor* script (run with *–cytosine_report and –CX* options) or, alternatively, by running the *coverage2cytosine* module. Cgmap [21] is another popular format for working with bisulfite data that provides sequence context and estimated DNA methylation level of any covered cytosines on the reference genome.

**Fig. 2:**
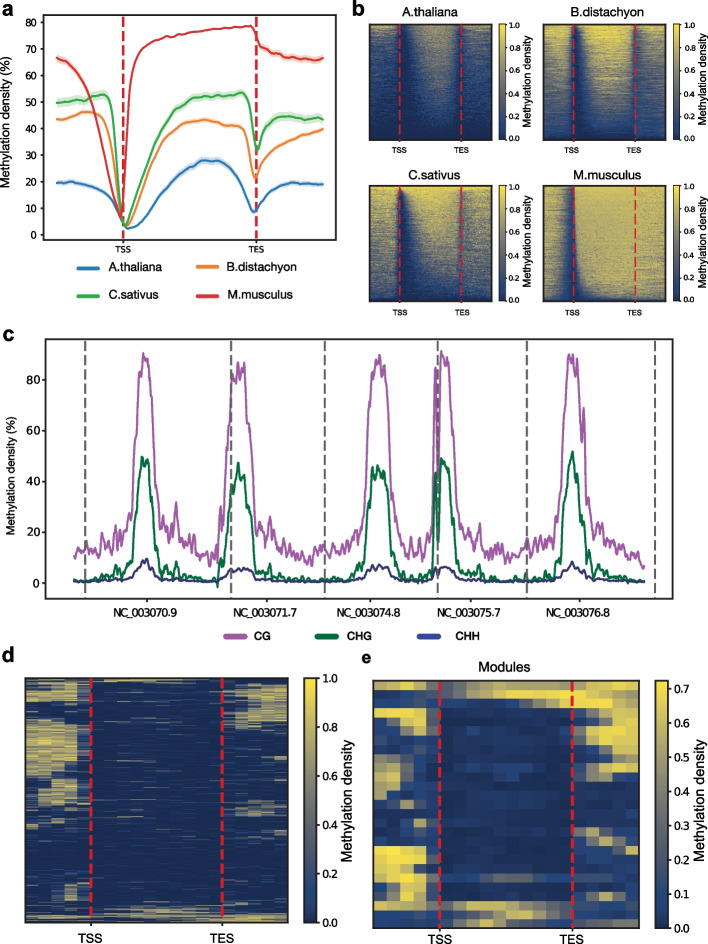
Comparative analysis of methylation patterns between species with BSXplorer. **a** Metagene visualization of CG methylation signal between different species. Confidence intervals are shown as shaded areas around lines. **b** Heatmap visualization of CG methylation in different species. Genes were ranked and sorted by overall methylation density. **c** Chromosome methylation levels for *Arabidopsis thaliana*. **d** Heatmap of *Brachypodium distachyon* CHG methylation context with genes clustered by their methylation pattern. **e.** Heatmap of *Brachypodium distachyon* CHG methylation context with genes split into modules, capturing dominant methylation patterns

BSXplorer offers visualization capabilities for generating average methylation coverage signal profiles over genomic regions of interest and heatmaps showing the overall signal pattern i.e., illustrating the enrichment of each region across the genome using colour gradients (Fig. 2, Additional file 1: Supplemental Data, sections II (c) and III). The coverage data for a given methylation context is then subjected to a normalization procedure via binning, which facilitates the comparison of regions of variable sizes (e.g., metagenes: gene bodies, transposable elements, etc.). Once the coverage signal is spilt into equal intervals, average density values for each interval are computed and visualised. To improve user experience and enhance data visualisation BSXplorer supports a) smoothing of the methylation profile plot with Savitzky–Golay filter [42], as well as estimation of a confidence interval for the standard deviation in order to quantify variability among methylation profiles (Fig. 2a), and b) exploration of DNA methylation variability between samples with violin and box plots (see Fig. 1 and Additional file 1: Supplemental Data). To allow for a comparison of methylation profiles in genomes of different species and varying sizes, BSXplorer provides a substantial flexibility in selection of parameters to specify a metagene, including minimal gene length, flank region length, number of bins to split genes and/or flanking regions.

To facilitate exploration of fundamental biology, BSXplorer provides a methylation signal density heatmap representation of the data, exhibiting the signal strength pattern of a high-throughput experiment over a set of genomic intervals (Fig. 2b). The ranking of regions is determined by the overall methylation level of genes, calculated by averaging of the mean densities of methylated cytosines (for a methylation context of interest) in a bin across all the bins in a particular gene. In order to identify the main patterns, we employed additional clustering methods on the methylation profiles. Heatmaps can become complex and difficult to interpret, particularly in cases where the methylation signal is not prominent (Fig. 2d,e and Additional file 1: Supplemental Data II (c)). To explore a particular group of regions that share a common motif, they can be exported and analyzed separately outside of BSXplorer.

Also, BSXplorer can graphically display methylation levels along chromosomes (Fig. 2c). Thus, each chromosome is tiled into non-overlapping regions of a specified length and average methylation density is computed and plotted.

Gene body methylation (GbM) [5] is an epigenetic mark found in animal and plant genomes where gene exons are methylated only in the CG context, as opposed to CHG and CHH contexts. In plants, where CG methylation is passed down through generations, GbM can affect up to 60% of genes in some species. The hypothetical functions of GbM, such as its potential role in regulating and stabilizing expression, preventing aberrant transcription, improving the fidelity of intron splicing [5], and its role in adaptation [13, 43], are currently being actively researched. Typically, GbM genes are defined statistically using a probabilistic approach as described in [44] and implemented in BSXplorer (Additional file 1: Supplemental Data, section III (b)).

Lastly, BSXplorer allows for the comparison of biological replicates, experiments, and species in a whole-genome mode, as well as for groups of regions-of-interest for all three methylation contexts (Additional file 1: Supplemental Data, sections III (b) and III (c)).

The availability of API and the ability to export results in different formats, such as TSV and RDS, makes it simple to incorporate BSXplorer routines into analytical pipelines (see Additional file 1: Supplemental Data for examples). In addition, the tool supports generation of hypertext reports, including interactive versions of plots.

BSXplorer is a highly versatile and user-friendly tool that offers an extensive range of features (see Table 1 for comparison with other solutions for methylation data analysis). It is especially relevant for non-model organisms and applications in crop science, plant biology, ecology and evolution. Typically, researchers working in these fields encounter objects that lack high-quality reference genomes and annotations of genomic elements, necessitating de novo chromosome-level genome assembly, gene prediction, and repetitive element discovery. Thus, researchers in these areas are faced with an unknown entity and exploratory data analysis and visual methylation profiling play a crucial role. Additionally, flexibility in selecting instruments for DMR/DMC detection is essential since many tools, especially if run in out-of-the-box mode, are primarily designed to work with classic model organisms. Furthermore, functional genomics analyses, such as GSEA, are typically performed outside of methylation data processing suites since they are based on computationally demanding orthologous gene mapping. When working with non-model organisms, it may be necessary to design a comprehensive solution (e.g., msPIPE or EpiDiverse). In such cases, we advocate for employing either Nextflow or Snakemake-based pipelines, which provide essential flexibility and versatility.

**Table 1:** Overview of BSXplorer features and comparison with other tools

	BSXplorer	MethGET	ViewBS	RnBeads	msPIPE	MethylC-analyzer
All-in-one						
BS-seq data analysis solution	No	No	No	Yes	Yes	Yes
CLI	Yes	Yes	Yes	Yes	No	No
API	Yes	No	No	Yes	No	No
Array support	No	No	No	Yes	No	No
Methylation data input format	Bismark (bedGraph, cov, CX_report), BS-Seeker2	BS-Seeker2, Bismark, BSMAP, methylpy, METHimpute	Bismark (CX_report), BSSeeker, BRAT	Array data, BS-seq fastq	BS-seq fastq	BS-Seeker, Bismark, BSMAP, METHImpute
Metagene line/heatmap plots	Yes	Metagene only	Yes	Yes	No	Yes
Genome annotation format	BED, GFF/GTF, custom	GTF	BED	Inbuilt set of human, mouse, rat genome annotations	Online access to USCS genomes	GTF
Regions-of-interest support	Yes	No	Yes	Yes	No	No
Intraspecies comparison	Yes	No	Yes	No	No	No
Data export for downstream analysis	Yes	No	Yes (RDS format)	No	Yes	Yes
Chromosome-level methylation	Yes	No	Yes	Yes	Yes	Yes
Comparative analysis of methylation profiles in different types of genomic elements	Yes	No	Yes	Yes	No	No
Comparative analysis of methylation profiles between experimental samples	Yes	Yes	Yes	Yes	No	Yes
Clustering of methylation profiles within the same experimental sample	Yes	No	No	No	No	No
Hypertext report generation	Yes	No	No	Yes	No	No
Non-model organism analysis support	Yes	Yes	Yes	No	Only USCS genomes	Yes
Genic DNA methylation pattern classification (i.e., Takuno & Gaut *P*_*CG*_ statistics)	Yes	No	No	No	No	No

To conclude, if a small-scale data perusal is required, BSXplorer can be used independently, or it can be integrated into these pipelines as needed (Additional file 1: Supplemental Data III (b)).

### Methylation data manipulation with BSXplorer

To demonstrate BSXplorer’s features and analytical capabilities, we selected a wide range of organisms diverse in many characteristics, including genome size, typical genome element length and methylation levels (see Additional file 1: Supplementary Information [38]).

The functionality of the BSXplorer package was demonstrated using whole genome BS-seq data from *Arabidopsis thaliana* (SRP014726, GSE39901) [45], cucumber—*Cucumis sativus* (SRP072226, GSE79526) [11], stiff brome—*Brachypodium distachyon* (SRP017401) and mouse (SRP013703, GSE57230) [46]. The choice of datasets facilitates intra-species comparisons of methylation profiles and demonstrates its seamless applicability to both classical model and non-model organisms (Additional file 1: Supplemental Data, sections II and III). Furthermore, to showcase the package we provide several moderate-sized datasets and the BSXplorer analysis workflow available on Zenodo [38–40].

BSXplorer provides excellent graphical analysis of sequencing data (see Fig. 2), encompassing profiling of average methylation levels in sites such as gene bodies, TEs, exons, etc. and along chromosomes, production of density heatmaps to demonstrate the strength of the signal in areas of interest, and distribution of methylation levels between samples.

BSXplorer tool allows researchers to compare and contrast methylation patterns in various species, including both model and non-model organisms. This feature is demonstrated using WGBS data generated to understand the resistance mechanisms of the "Misugi" cultivar of Japanese mustard spinach (*Brassica rapa* subsp. *perviridis*) against white rust, which is a fungal infection caused by *Albugo candida* [47] (Additional file 1: Supplemental Data, sections III (b), (c)).

In addition to comparing different conditions/methylation contexts within the same organism/experiment, BSXplorer enables evaluation of methylation signals across different species. In the past decade, it has become evident that genome-wide DNA methylation patterns differ greatly across species [11, 12]. Methylation patterns in flowering plants are very similar, with methylated cytosines being detected in all sequence contexts, while CG methylation is more prevalent in animals. For example, as shown in Fig. 2a,b and in Additional file 1: Supplemental Data, section III (c), all three flowering plants exhibited a characteristic peak in the body of protein-coding genes, a phenomenon first observed genome-wide in *Arabidopsis*. In mice, as expected, a slightly higher CG methylation was observed in the body of genes, and there was a depletion of methylation around transcriptional start sites, coinciding with CpG islands. Also, BSXplorer provides a special feature allowing comparison of gene methylation patterns within the same sample and group them using the hybrid dynamicTreeCut method [48]. The gene clusters are depicted on a heatmap in a similar way as methylation level profiles (Fig. 2d,e).

## Conclusions

BSXplorer is a tool developed to facilitate the graphical analysis of DNA methylation patterns in genomes, both at functional genomic elements and user-defined regions, with unmatched data processing speed. It offers both API and CLI, allowing for seamless integration into data analysis workflows and scripts. The tool enables comparison of methylation signals between different contexts, samples, and species at functional genomic elements and regions of interest. Furthermore, the package categorizes genome regions based on methylation status using a probabilistic approach. BSXplorer also generalises on BS-seq signal profiles to produce gene modules exhibiting similar methylation signatures for thorough exploration at a functional level. Overall, BSXplorer is a lightweight and flexible instrument that facilitates explorative analyses of DNA methylation patterns in genomes of model and non-model organisms in an efficient way.

## Availability and requirements

Project name: BSXplorer.

Project home page: https://github.com/shitohana/BSXplorer/

Archived version: https://zenodo.org/records/10702272

Operating system(s): Platform independent.

Programming language: Python (version 3.9.0 or higher).

Other requirements: Bismark methylation caller suite; Python libraries biopython (v. 1.81), dynamicTreeCut (v. 0.1.1), fastcluster (v. 1.2.6), Jinja2 (v. 3.1.2), matplotlib (v. 3.8.0), numba (v. 0.58.1), numpy (v. 1.26.3), pandas (v. 2.2.0), plotly (v. 5.18.0), polars (v. 0.20.7), progress (v. 1.6), pyarrow (v. 13.0.0), pyreadr (v. 0.4.9), scikit-learn (v. 1.4.0), scipy (v. 1.12.0), seaborn (v. 0.13.2) License: GNU GPLv3.

Any restrictions to use by non-academics: none.

### Supplementary Information

**Additional file 1.** Supplementary Materials, Figures, Package Tutorial and Examples of Usage.

## Data Availability

The datasets supporting the conclusions of this article are available at the Zenodo repository, https://zenodo.org/records/10702195 and https://zenodo.org/records/10702204 ([39, 40]).

## Abbreviations

API: Application programming interface
BS-seq: Bisulfite sequencing
ChIPseq: Chromatin Immunoprecipitation Sequencing
DMR: Differentially methylated regions
DMC: Differentially methylated cytosines
CLI: Command line interface
EWAS: Epigenome-wide association studies
GWAS: Genome-wide association studies
GbM: Gene body methylation
RAM: Random access memory
RNAseq: RNA-sequencing
