# Supplementary material for: BSXplorer: analytical framework for exploratory analysis of BS-seq data
Source: BMC Bioinformatics. 2024 Mar 4;25:96. doi: 10.1186/s12859-024-05722-9 (PMC10913661; doi:10.1186/s12859-024-05722-9)
Supplement: Supplementary file 1 — Additional file 1. Supplementary Materials, Figures, Package Tutorial and Examples of Usage. [file 12859_2024_5722_MOESM1_ESM.pdf]

Supplementary Information for
“BSXplorer: analytical framework for exploratory analysis of BS-seq data.”
Konstantin Yuditskiy, Igor Bezdvornykh, Anastasiya Kazantseva, Alexander Kanapin, Anastasia Samsonova

BSXplorer analysis workflow

BSXplorer is a powerful, yet lightweight tool for analysis of bisulfite sequencing data which offers a range of features such as the ability to profile methylation levels in metagenes or user-defined loci using both line plots and heatmaps. It also allows for comparative analyses of methylation patterns across experimental samples and species, and can identify gene modules that share similar methylation signatures at functional elements. Additionally, BSXplorer enables the generation of summary statistics charts for quick and easy data visualization. While BSXplorer was originally designed for non-model organism research with a specific emphasis on plants biology and agrigenomics, it can also be used for exploratory data analysis in model organisms.

BSXplorer is implemented in Python (3.9 or higher). This vignette will guide you through the details of the BSXplorer workflow, highlighting key features, with a particular focus on generation of HTML-reports, its integration within Jupyter notebooks (API) and fine-tuning of essential parameters.

I. Installation

BSXplorer can be installed by running the following command on your system.

```
pip install bsxplorer
```

This command installs the package and dependencies, as well as adds the corresponding environmental variables to the system's PATH, enabling command-line access to the package, thus facilitating seamless integration into the user's computational environment.

II. Getting started

The analysis process can be divided into four steps as follows:

a. Import annotation file
b. Read cytosine report file (e.g., Bismark output)
c. Get results

The example analysis is prepared for *Arabidopsis thaliana* chromosome 3 (NC_003074.8).
The test data can be downloaded from the Zenodo repository via this link:
https://zenodo.org/records/10702195

a. Import the annotation file

To import genome annotation from file use BSXplorer function **bsxplorer.Genome.from_gff()**. The parameters provided to the function include a path to the annotation file in GFF format. As GFF format provides richer annotation and not readily available for non-model organisms, one could also use annotations in GTF/BED formats.

```
genome = bsxplorer.Genome.from_gff("arath_genome.gff")
```

Next, the annotation is filtered to extract the genomic regions of interest. E.g., this can be achieved with **gene_body()** function from the class **Genome,** to filter only genes from annotation. Notably, the function takes in arguments to identify the flanking regions, e.g., **flank_length**, as well as a minimum length of a gene to be included in the analysis (**min_length**).

```
genes = genome.gene_body(flank_length=2000, min_length=3000)
```

Upon completion of these steps, the annotation file can be combined with the methylation data from the cytosine report, making it possible to perform analyses of DNA methylation patterns within particular genomic contexts.

b. Read the cytosine report file

To analyse the cytosine report and carry out metagene analysis, BSXplorer offers the **Metagene** class. In order to read the Bismark's methylation_extractor output file, the function **from_bismark()**of the Metagene class should be utilised. This function requires the file path and the annotation file mentioned above as mandatory parameters. Additionally, users can specify parameters such as the number of windows for flanking regions and the gene body (**up_windows, gene_windows, down_windows**) as shown below:

```
metagene = bsxplorer.Metagene.from_bismark("arath_example.txt", genes, up_windows=100,
body_windows=200, down_windows=100)
```

Following this, one has to wait for the file reading process to be completed, as depending on the file size it may take a while. Once the file reading is done, the data can be visualised and analysed.

c. Get results

1. Single sample

Depending on the analyses goals it may be required to filter the cytosine report file to extract information on the methylation context of interest as well as on strand attribution of a methylation event. This is achieved by using the **Metagene.filter()** function:

```
filtered = metagene.filter(context="CG", strand="+")
```

The smoothened line plot, showing the average methylation density in the metaregion of interest (e.g., gene body, plus upstream and downstream regions of desired length), can be generated with the

Metagene.line_plot().draw_mpl() function, which returns the **matplotlib.Figure** object. The desired degree of signal smoothing is determined by the *a priori* specified number of windows.

```
filtered.line_plot().draw_mpl(smooth=10)
```

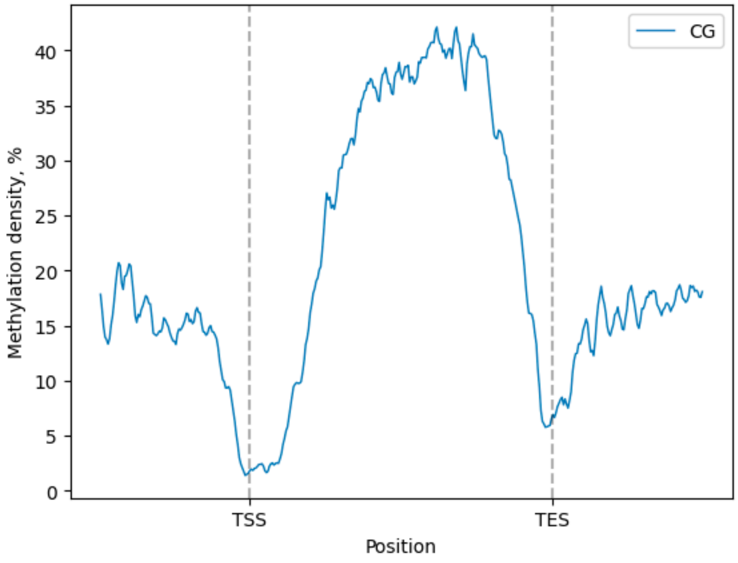

Alternatively, a heatmap representation of the methylation signal density is made available by application of the **Metagene.heat_map().draw_mpl()** method, where heatmap resolution (i.e., **ncol** and **nrow** parameters) is adjustable by specifying the arguments of the **heat_map()** function.

```
filtered.heat_map(100, 100).draw_mpl()
```

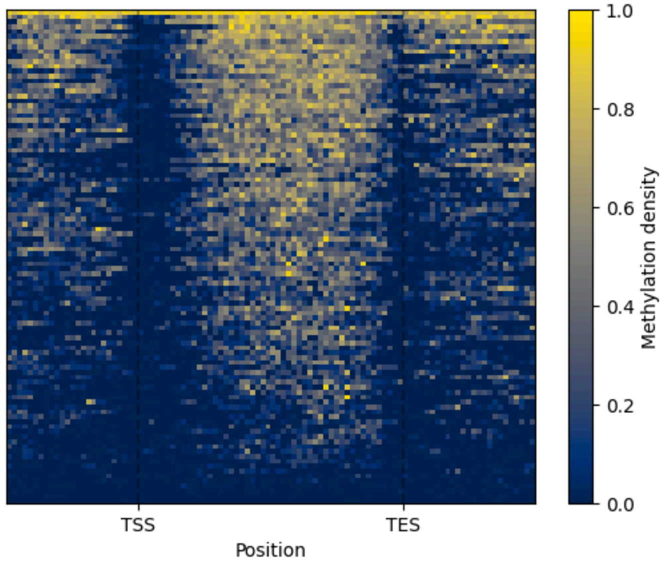

2. Contrasting methylation profiles in different species

Start with import of genome annotation data for species of interest.

```
arath_genes = bsxplorer.Genome.from_gff(“arath_genome.gff”).gene_body(min_length=0)
```

```
bradi_genes = bsxplorer.Genome.from_gff("bradi_genome.gff").gene_body(min_length=0)
mouse_genes = bsxplorer.Genome.from_gff("musmu_genome.gff").gene_body(min_length=0)
```

Next, read in cytosine reports for each sample separately:

```
arath_metagene = bsxplorer.Metagene.from_bismark("arath_example.txt", arath_genes,
200, 400, 200)
bradi_metagene = bsxplorer.Metagene.from_bismark("bradi_example.txt", bradi_genes,
200, 400, 200)
musmu_metagene = bsxplorer.Metagene.from_bismark("musmu_example.txt", mouse_genes,
200, 400, 200)
```

To perform comparative analysis, initialize the **bsxplorer.MetageneFiles** class using metagene data in a vector format, where **labels** for every organism are provided explicitly.

```
files = bsxplorer.MetageneFiles([arath_metagene, bradi_metagene, musmu_metagene],
                                labels=["AraTh", "BraDi", "mouse"])
```

Next, apply methylation context and strand filters to the input files:

```
filtered = files.filter("CG", "+")
```

Then, a compendium of line plots to guide a comparative analyses of methylation patterns in different species is constructed:

```
filtered.line_plot().draw_mpl(smooth=50)
```

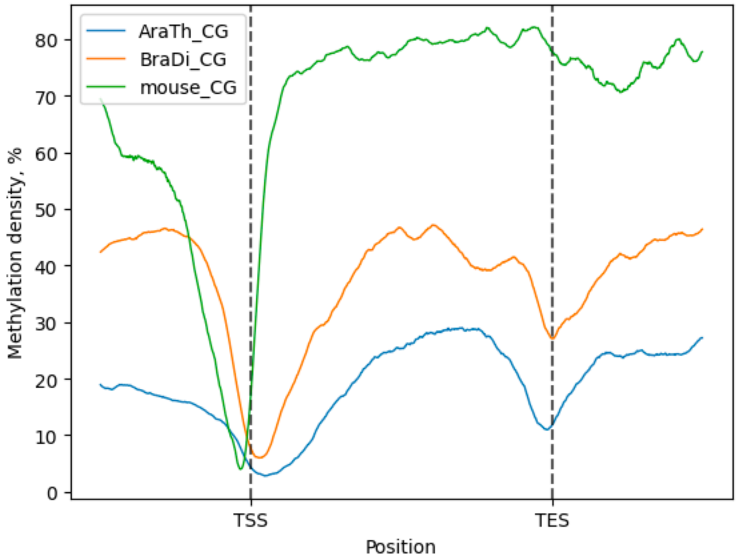

The line plot representation may be further supplemented by a heatmap:

```
filtered.heat_map(100, 100).draw_mpl()
```

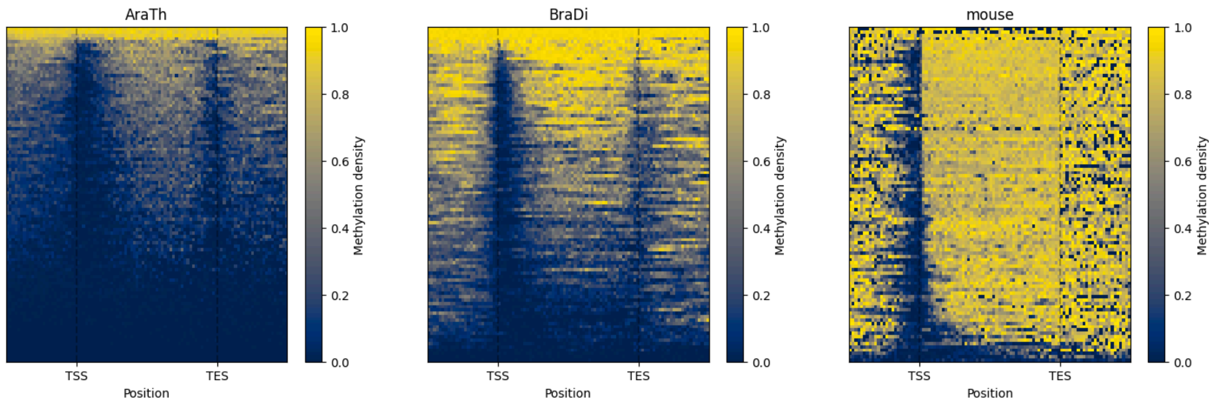

To examine and highlight differences in methylation patterns between different organisms, summary statistics is made available in a graphical format.

```
filtered.violin_plot()
filtered.box_plot()
```

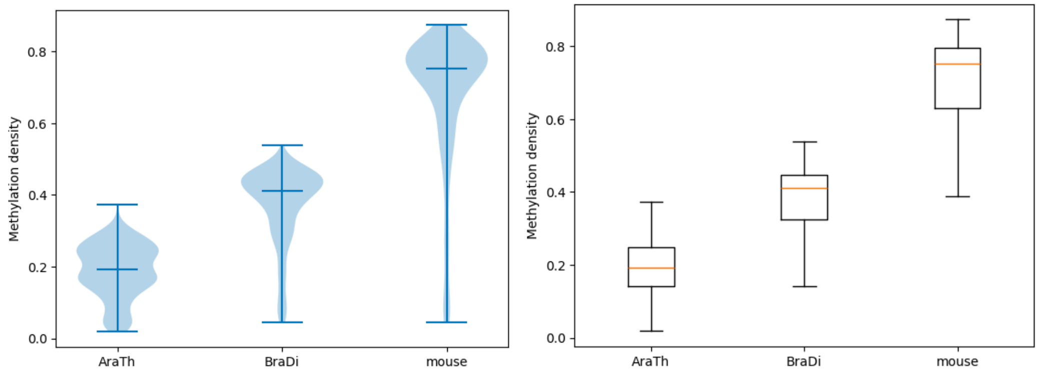

3. Clustering of methylation patterns and module discovery

BSXplorer allows for discovery of gene modules characterised with similar methylation patterns. Importantly, as this type of analysis involves hierarchical clustering, it can be resource-hungry and time-consuming. Therefore, it is not recommended to use more than 20 bins per gene, when testing the package.

```
Import bsxplorer

arath_genome = bsxplorer.Genome.from_gff("arath_genome.gff")
arath_genes = arath_genome.gene_body(min_length=0, flank_length=2000)


arath_metagene = bsxplorer.Metagene.from_bismark(
    "arath_example.txt", arath_genes,
```

```
    up_windows=5, body_windows=10, down_windows=5
)
```

Once the data was filtered based on methylation context and strand, one can use the **.clustering()** method. Set the **count_threshold** parameter to define the minimum count of cytosine reads to consider when producing gene clusters. The resulting **Clustering** object contains an ordered list of clustered genes and their visualisation in a form of a heatmap.

```
Arath_filtered = arath_metagene.filter(context="CG", strand="+")
arath_clustered = arath_filtered.cluster(count_threshold=5, na_rm=0).all()
```

To visualise the clustered genes, use the **.draw()** method. This function accepts the same set of parameters as the standard **.heat_map** method shown above in section B.

```
arath_clustered.draw_mpl()
```

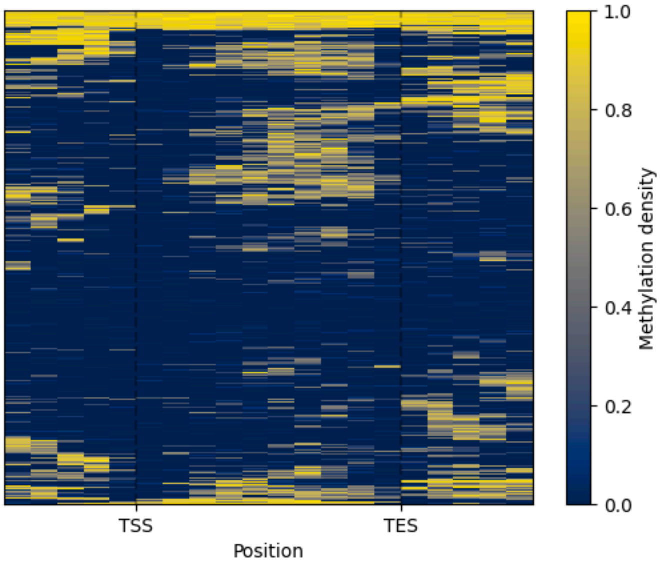

To identify gene modules that exhibit similar methylation patterns apply the **.modules()** function of the **Clustering** class. This method relies on the dynamicTreeCut algorithm to find modules.

```
Arath_modules =
arath_filtered.cluster(count_threshold=5, na_rm=0).kmeans(n_clusters=5)
arath_modules.draw_mpl()
```

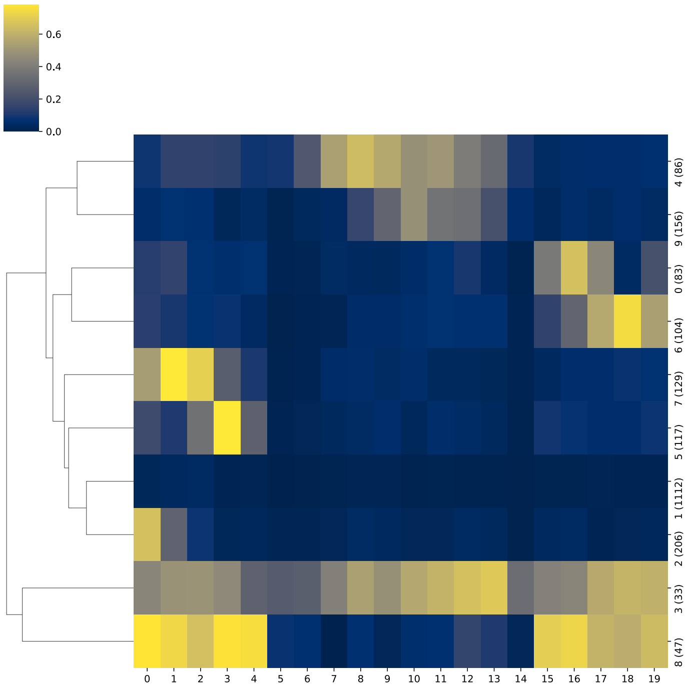

To explore BSXplorer features in full and to keep track of the recent developments follow the following link to the package's GitHub repository:
https://github.com/shitohana/BSXplorer

III. Exploratory data analyses

The objective of this section is to showcase how BSXplorer can be utilized to perform exploratory data analyses (EDA), with a focus on BS-seq data produced in non-model organisms. Such organisms may lack reference genome sequence and canonical genome annotation available in public repositories (e.g., UCSC or Ensembl).

The data used in the scenarios below can be downloaded from the Zenodo repository via this link: https://zenodo.org/records/10702204 .

Here are three scenarios for analyzing BS-seq data:

a. EDA of BS-seq data obtained from non-model organisms in console mode;
b. EDA of BS-seq data from non-model organisms in different conditions; and
c. EDA of BS-seq data from different non-model organisms (intra-species).

a. EDA of BS-seq data generated from non-model organism

BSXplorer is a tool that is specifically designed for easy EDA and visualization of bisulfite sequencing data. It is particularly useful for non-model organisms such as commercially valuable crops and other plants, where often there is no reference material available. Researchers in such cases may only have access to

an assembled genome and partially or spuriously assembled CDS from publications and associated genomic projects. To demonstrate its capabilities, we will use *Brassica rapa* subsp. *perviridis* WGBS dataset [1] as an example.

The analysis begins with the initialization of the ***Genome*** object, which stores information about the annotation of genomic regions and their coordinates**.**

```
genome = bsxplorer.Genome.from_custom(
    "genomic_id_ncbi.tsv",
    chr_col=0, type_col=1, start_col=2, end_col=3, strand_col=4, id_col=5,
has_header=True
).gene_body(min_length=0, flank_length=2000)
```

Importantly, the method mentioned above can accept genomic annotation data in both GFF/GTF format and simple tab-delimited format. To create a ***Genome*** object, a user needs to provide a minimal set of columns that include genomic coordinates such as chromosome, strand, start, and end positions. In the example code provided, a ***Genome*** object was initialized using custom annotation by using the ***from_custom()*** constructor. During initialization, the corresponding column indices are specified. Once the **Genome** object is initialized, it is filtered by region type (gene), minimum region length, and coordinates of flanking regions are created using the ***.gene_body()*** method to prepare it for further use.

The bisulfite sequencing data can be imported as a bedGraph file, as coverage data or in the form of a methylation report. The latter is generated by Bismark suite's ***methylation_extractor*** tool.

The ***Metagene*** object is initialized with the dedicated constructor as shown below:

```
metagene = bsxplorer.Metagene.from_bismark(
    "DRR336466.CX_report.txt.gz", genome,
    up_windows=250, body_windows=500, down_windows=250
)
```

To generate a simple line plot to visualize DNA methylation patterns in gene regions the BSXplorer's ***.line_plot()*** is applied. Then to render the figure the ***.draw_mpl()*** method of is used.

```
metagene.line_plot(stat="mean").draw_mpl(
    confidence=.99,
    major_labels=["", ""],
    minor_labels=["-2000bp", "Gene body", "+2000bp"]
)
```

In this context, the ***stat*** parameter determines the statistical method used to summarize the data in each bin when computing line values. For example, by setting the ***stat*** parameter to "mean," the mean value will be used as the summary function. The ***confidence*** parameter is used to depict confidence intervals

for a confidence probability of 0.99. To remove the default labels (i.e., TSS/TES) at gene boundaries, the ***major_labels*** parameter can be set to an empty list. The ***minor_labels*** parameter can be used to set labels for the flanking and gene body regions. In this example, the parameter is set to ["-2000bp", "Gene body", "+2000bp"].

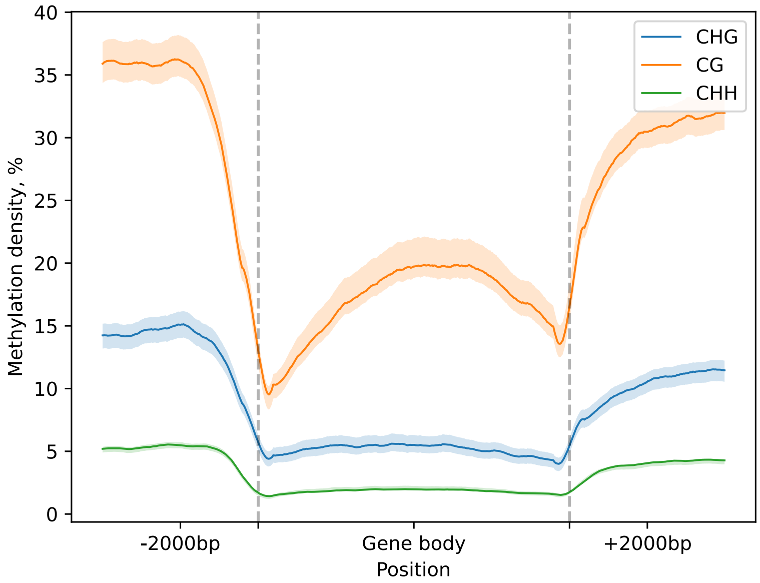

The metagene object to can be subsetted by methylation context and strand to make a customized heatmap representation (with a ***.heat_map()*** function) of methylation pattern in/around gene regions.

```
filtered = metagene.filter(context="CG", strand="-")

filtered.heat_map(ncol=100, nrow=100).draw_mpl(
    major_labels=["", ""],
    minor_labels=["-2000bp", "Gene body", "+2000bp"]
)
```

The parameters ***ncol*** and ***nrow*** set dimensions for the heatmap to display.

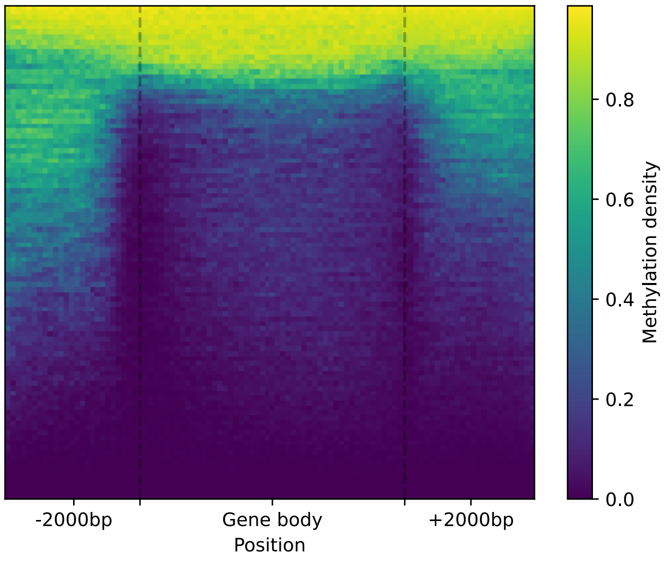

In cases where a heatmap is too cluttered, clustering genes can generate a clearer representation of the heatmap.

```
kmeans = filtered.resize(20).cluster(count_threshold=5,
na_rm=0).kmeans(n_clusters=10)
kmeans.draw_mpl()
```

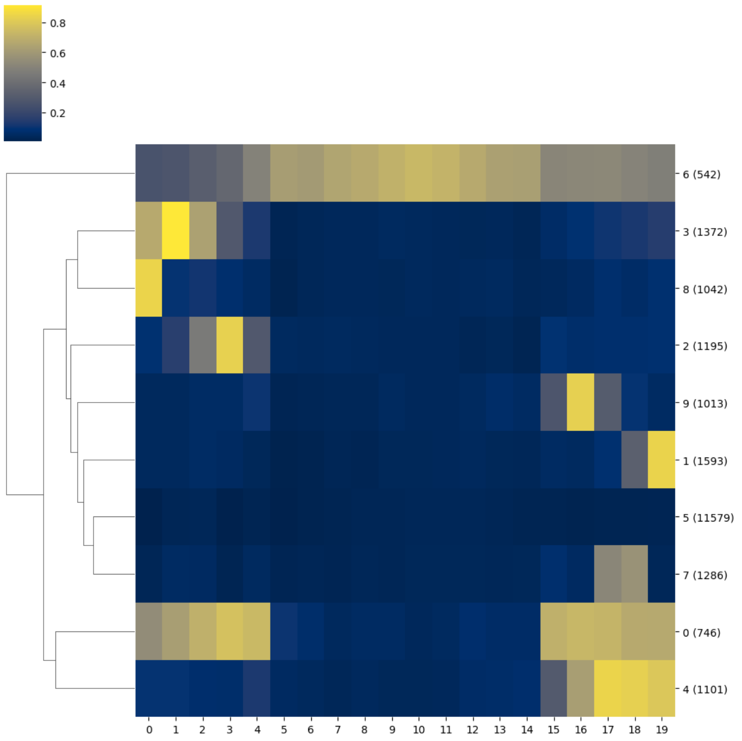

To facilitate further exploration of the data via, for instance, functional enrichment analyses the tab-delimited list of regions and the associated module IDs can be exported to a file, as shown below:

```
kmeans.save_tsv("kmeans_labels.tsv")
```

BSXplorer allows a user to visualize the overall methylation levels of chromosomes using the corresponding ***ChrLevels*** object:

```
levels = bsxplorer.ChrLevels.from_bismark("DRR336466.CX_report.txt.gz",
chr_min_length=10**6, window_length=10**6)

levels.draw_mpl(smooth=5)
```

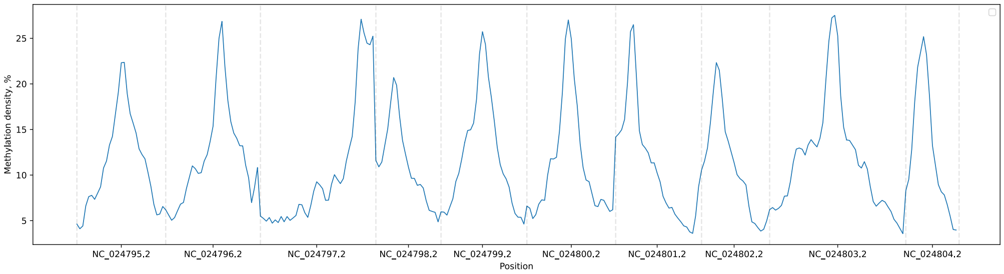

In a way that is similar to the ***Metagene*** method, the methylation data can be subjected to filtering to selectively display a methylation context that is of interest.

```
levels.filter(context="CG").draw_mpl(smooth=5)
```

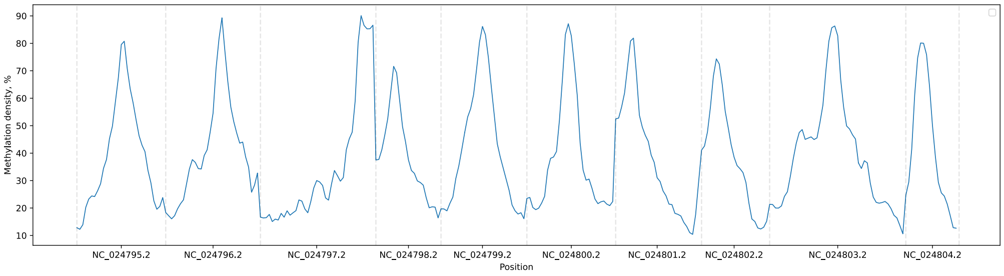

BSXplorer is a tool that provides various capabilities for the analysis, comparison, and visualization of methylation profiles for different samples and genomic elements. For instance, it can compare methylation patterns of genes and transposable elements in *Brassica rapa* subsp. *perviridis*.

```
annotation = bsxplorer.Genome.from_custom(
    "genomic_id_ncbi.tsv",
    chr_col=0, type_col=1, start_col=2, end_col=3, strand_col=4, id_col=5,
has_header=True
)

genes = annotation.gene_body(min_length=0, flank_length=500)
te = bsxplorer.Genome.from_gff("TE.gff").other("match", 0, flank_length=500)
```

To start the analysis, we first read the annotation and then select genes (***.gene_body***) and transposable elements (***.other(region_type="match")***) for further analysis. We then create an object using the ***MetageneFiles*** class that stores information about several metagenes.

```
metagenes = bsxplorer.MetageneFiles.from_list(
    ["DRR336466.CX_report.txt.gz", "DRR336466.CX_report.txt.gz"],
    [genome, te],
    up_windows=20, body_windows=40, down_windows=20,
    labels=["Genes", "TE"]
)
```

The paths to the files, annotations, the number of windows on each region, and labels for plotting are specified in the ***.from_list*** constructor. Also, as shown previously, the object is filtered by DNA strand and methylation context prior to plotting.

```
filtered = metagenes.filter(context="CG", strand="-")

labels_settings = dict(major_labels=["", ""], minor_labels=["-500bp", "Body",
"+500bp"])
filtered.line_plot().draw_mpl(smooth=5, confidence=.95, **labels_settings)

filtered.heat_map(10, 20).draw_mpl(**labels_settings)
```

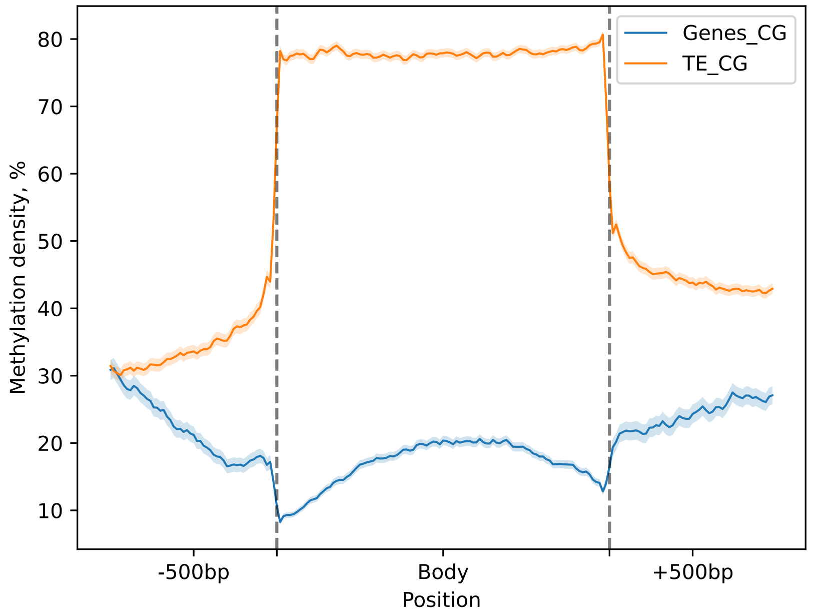

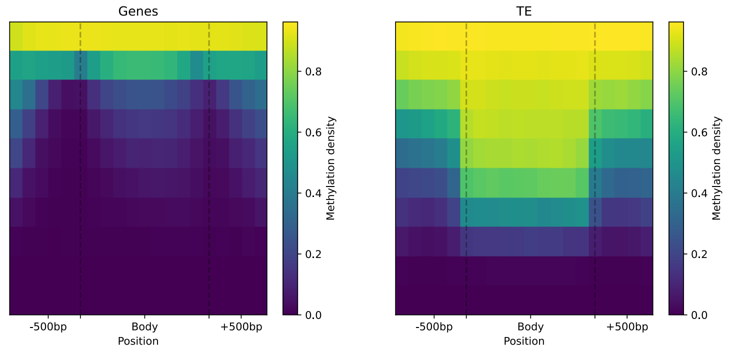

The ***MetageneFiles*** object has ***.box_plot()*** and ***.violin_plot()*** functions for box plot and violin plot graphs. The ***.trim_flank()*** method analyzes methylation of the body of the region.

```
filtered.trim_flank().box_plot()

filtered.trim_flank().violin_plot()
```

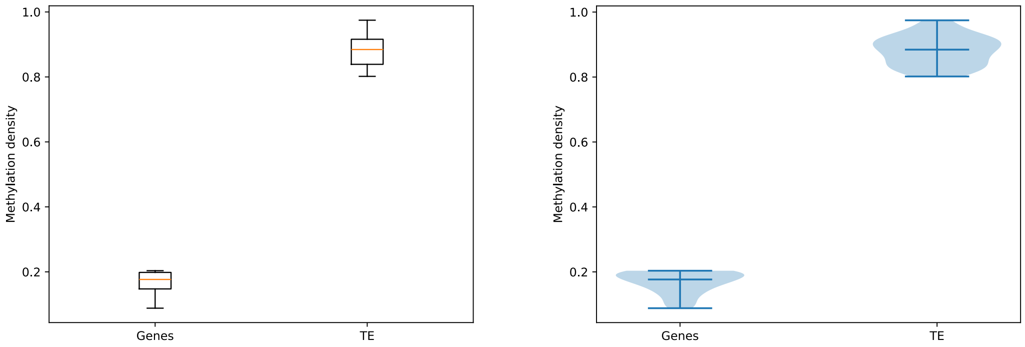

b. EDA of BS-seq data generated from non-model organisms in different conditions

BSXplorer allows for the categorization of regions based on their methylation level and density. This is done by assuming that cytosine methylation levels follow a binomial distribution, as explained in Takuno and Gaut's work (please refer to [2, 3] https://doi.org/10.1073/pnas.1215380110 for details). The genes are then divided into three categories, BM (body-methylated), IM (intermediately-methylated) and UM (under-methylated), based on their methylation levels in the CG context using the following formula.

$$CG < P_{CG}, \qquad CHG/CHH > 1 - P_{CG}$$
$$P_{CG} \leq CG < 1 - P_{CG}, \qquad CHG/CHH > 1 - P_{CG}$$
$$CG/CHG/CHH > 1 - P_{CG}$$

The same rationale may be applied to other methylation contexts, as BSXplorer can produce P_{CHG} and P_{CHH} for CHG sites and CHH sites, respectively.

Although BSXplorer has full functionality for analysis within PythonAPI, we will demonstrate the categorization of regions and their visualization using a console script in this example.

```
bsxplorer-category -o TE_Cat_Report --dir TE_Cat -u 50 -d 50 -b 100 -S 10 --
ticks \\-500bp \\ Body \\ \\+500bp -V 50 -H 50 --save_cat te_conf.tsv
```

The API version of the above command string is presented in the code snippet below:

```
annot = bsxplorer.Genome.from_gff("TE.gff")
annot = annot.other(region_type="match", flank_length=500, min_length=200)

mock_merged = merge_replicates(
    ["DRR336466.CX_report.txt.gz", "DRR336467.CX_report.txt.gz"],
    report_type="bismark"
)
inf_merged = merge_replicates(
    ["DRR336468.CX_report.txt.gz", "DRR336469.CX_report.txt.gz"],
    report_type="bismark"
)
```

```
p_value_kwargs = dict(
    genome=annot,
    methylation_pvalue=.05
)

mock_binom = bsxplorer.BinomialData.preprocess(
    mock_merged.name, report_type="parquet", min_coverage=2
)
mock_pstat = mock_binom.region_pvalue(**p_value_kwargs)

inf_binom = bsxplorer.BinomialData.preprocess(
    inf_merged.name, report_type="parquet", min_coverage=2
)
inf_pstat = inf_binom.region_pvalue(**p_value_kwargs)


categorise_kwargs = dict(
    context="CG", p_value=.05, min_n=5
)

# Returns tuple with (BM, IM, UM) ids
mock_cat = mock_pstat.categorise(**categorise_kwargs, save="mock")
inf_cat = inf_pstat.categorise(**categorise_kwargs, save="mock")

metagene_kwargs = dict(
    genome=annot,
    up_windows=50, body_windows=100, down_windows=50,
)
mock_metagene = bsxplorer.Metagene.from_binom(mock_binom.path,
**metagene_kwargs)
inf_metagene = bsxplorer.Metagene.from_binom(inf_binom.path, **metagene_kwargs)

# e.g. for CG BM
metagenes = bsxplorer.MetageneFiles([
    mock_metagene.filter(context="CG", genome=mock_cat[0]),
inf_metagene.filter(context="CG", genome=mock_cat[0])
], ["Mock", "Infected"])

tick_kwargs = dict(
    major_labels=["", ""],
    minor_labels=["-500bp", "Body", "+500bp"]
)

metagenes.line_plot().draw_mpl(smooth=10, **tick_kwargs)
metagenes.heat_map(50, 50).draw_mpl(**tick_kwargs)
```

A user can obtain a complete list of parameters by using the command ***bsxplorer-category --help***. The configuration file has the following structure

*This header is for demonstration purposes only and it **should not be** included in the conf.tsv.*					
Sample group	Path to report	Path to genome	Flank length	Minimal length	Region_type
Misugi_mock	DRR336466.CX_report.txt.gz	TE.gff	500	0	match
Misugi_mock	DRR336467.CX_report.txt.gz	TE.gff	500	0	match
Misugi_infected	DRR336468.CX_report.txt.gz	TE.gff	500	0	match
Misugi_infected	DRR336469.CX_report.txt.gz	TE.gff	500	0	match

The ***bsxplorer-category*** command imports Bismark's cytosine report file and performs a binomial test to determine the statistical significance of methylation at each site. The command uses the estimated error rate to conduct the test and generates corresponding p-values, which can be exported using the API. Additionally, the tool imports genomic annotation for each region in every context and calculates the P_{CG} (or P_{CHG} or P_{CHH}) value. Finally, it filters and classifies the regions into UM, BM and other classes based on

the results obtained.

The following plots were generated for the BM and UM regions (CG-context) as part of the HTML-report.

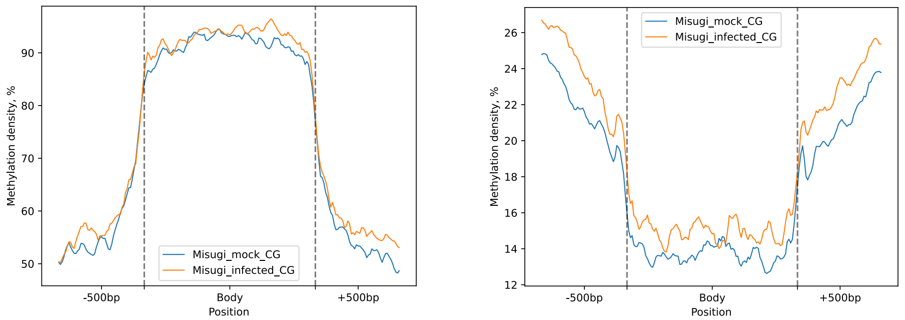

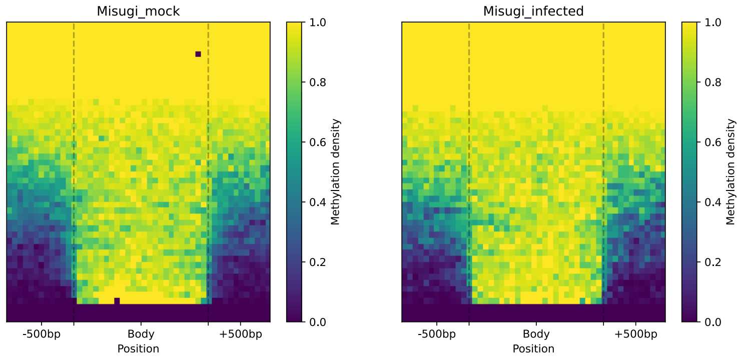

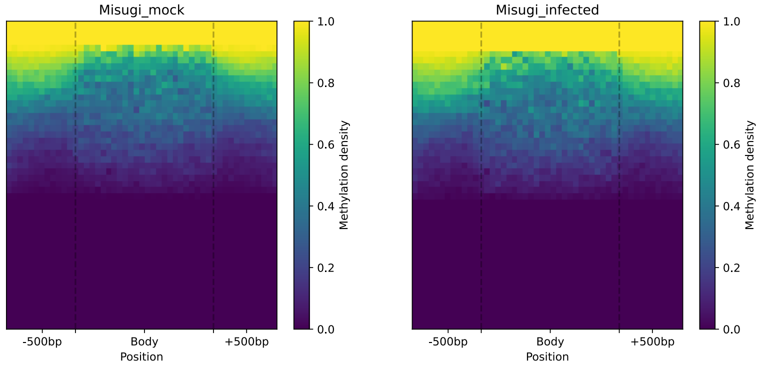

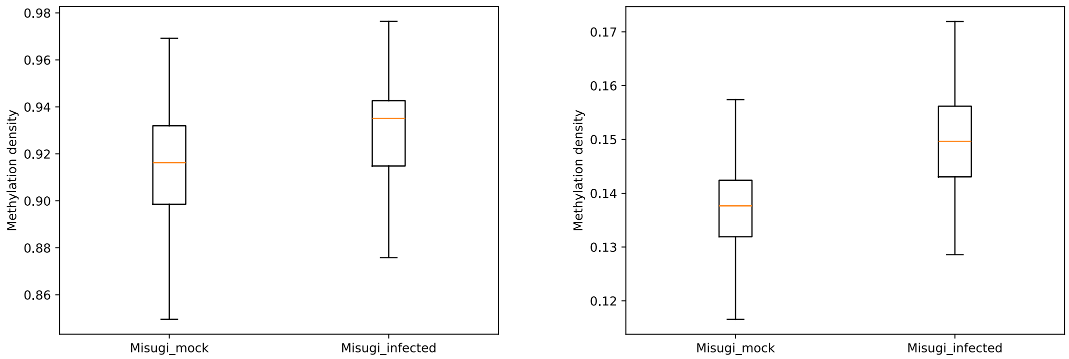

A thorough examination of methylation profiles associated with transposable elements in *Brassica rapa* subsp. *perviridis* has revealed a distinct group of genomic elements changing their methylation pattern from UM to BM in response to the white rust infection, caused by the fungus *Albugo candida.*

Using BSXplorer, the following section displays a practical approach to presenting these elements. The control and infected samples are referred to as "mock" and "inf" respectively. Here we assume that $\boldsymbol{P_{CG}}$ statistics which enables UM and BM classification has been computed in the previous steps of analyses.

After importing the bisulfite data for the transposons (Misugi_mockCG_UM.tsv and Misugi_infectedCG_BM.tsv), the sets are intersected, matched with the annotation file, and the resulting patterns are visualized and compared to other regions.

```
import polars as pl

mock_um = pl.read_csv("Misugi_mockCG_UM.tsv", separator="\t",
has_header=False)[:, 3].to_list()

inf_bm = pl.read_csv("Misugi_infectedCG_BM.tsv", separator="\t",
has_header=False)[:, 3].to_list()

mock_inf_up = list(set.intersection(set(mock_um), set(inf_bm)))

te = bsxplorer.Genome.from_gff("TE.gff").other("match", 0, flank_length=500)
```

The metagene is constructed using the mean statistics with the ***sumfunc="mean"*** parameter.

```
args = dict(genome=te, up_windows=50, body_windows=100, down_windows=50,
sumfunc="mean")
metagene_mock =
bsxplorer.MetageneFiles.from_list(["DRR336466.CX_report.txt.gz",
"DRR336467.CX_report.txt.gz"], labels=["mock-1", "mock-2"], **args)
metagene_inf = bsxplorer.MetageneFiles.from_list(["DRR336468.CX_report.txt.gz",
"DRR336469.CX_report.txt.gz"], labels=["inf-1", "inf-2"], **args)


metagene_mock = metagene_mock.merge()
metagene_inf = metagene_inf.merge()
```

The ***.merge()*** method of the ***MetageneFiles*** class is used to combine biological replicates. The following code snippet creates a clustergram for a selected subset of genomic regions of interest using the ***.dendrogram()*** function. The "q" argument is set to 0, which means that all regions are included in the resulting plot.

```
dendro_metagene =
bsxplorer.MetageneFiles([metagene_mock.filter(id=mock_inf_up),
metagene_inf.filter(id=mock_inf_up)], ["mock_up", "inf_up"])
dendro_metagene.dendrogram(q=0)
```

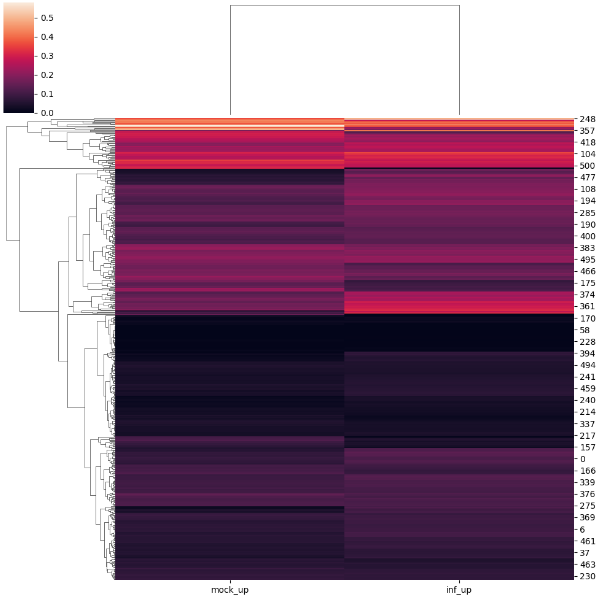

To generate a clustergram for the same set of transposons, where the methylation levels of biological replicates are presented independently, use the following set of commands:

```
metagene_mock =
bsxplorer.MetageneFiles.from_list(["DRR336466.CX_report.txt.gz",
"DRR336467.CX_report.txt.gz"], labels=["mock-1", "mock-2"], **args)
metagene_inf = bsxplorer.MetageneFiles.from_list(["DRR336468.CX_report.txt.gz",
"DRR336469.CX_report.txt.gz"], labels=["inf-1", "inf-2"], **args)
all_replicates = bsxplorer.MetageneFiles(
    metagene_mock.samples + metagene_inf.samples,
    metagene_mock.labels + metagene_inf.labels
)
all_replicates.dendrogram(q=.95)
```

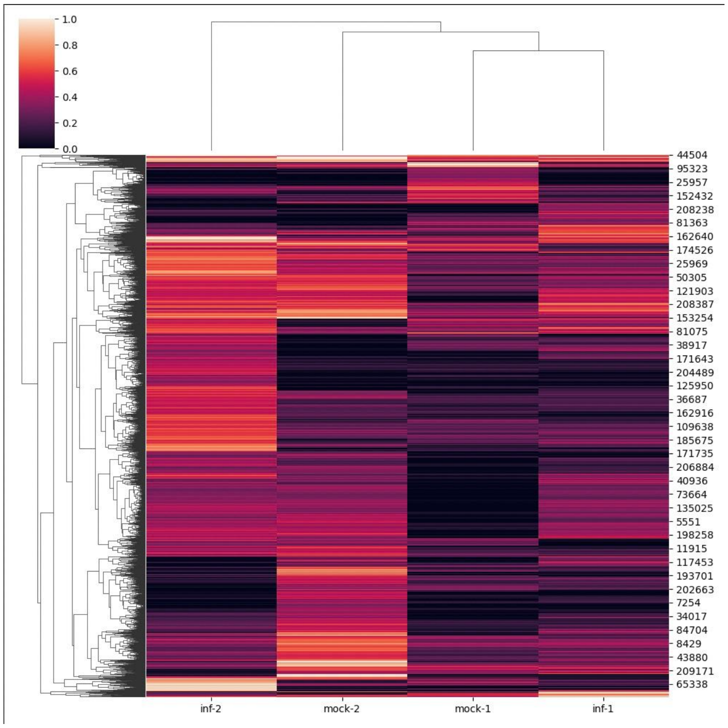

To visually compare the UM and BM regions that contain transposable elements with the remaining genomic regions that exhibit UM or BM patterns, we create a ***MetageneFiles*** object. This object enables us to generate line and box plots, as well as heatmaps that display methylation levels in the aforementioned classes.

```
metagenes = bsxplorer.MetageneFiles([metagene_mock, metagene_inf,
metagene_mock.filter(id=mock_inf_up), metagene_inf.filter(id=mock_inf_up)],
["mock_all", "inf_all", "mock_up", "inf_up"])
filtered = metagenes.filter(context="CG")

ticks = {"major_labels": ["", ""], "minor_labels": ["-500bp", "Body",
"+500bp"]}
filtered.line_plot().draw_mpl(smooth=10, **ticks)

filtered.heat_map(20, 20).draw_mpl(**ticks)
filtered.trim_flank().box_plot()
```

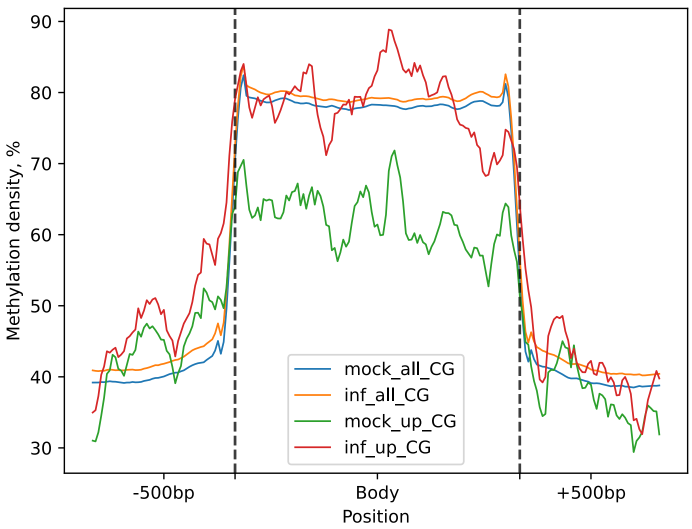

Methylation density, %
90
80
70
60
50
40
30
-500bp
Body
+500bp
Position
mock_all_CG
inf_all_CG
mock_up_CG
inf_up_CG

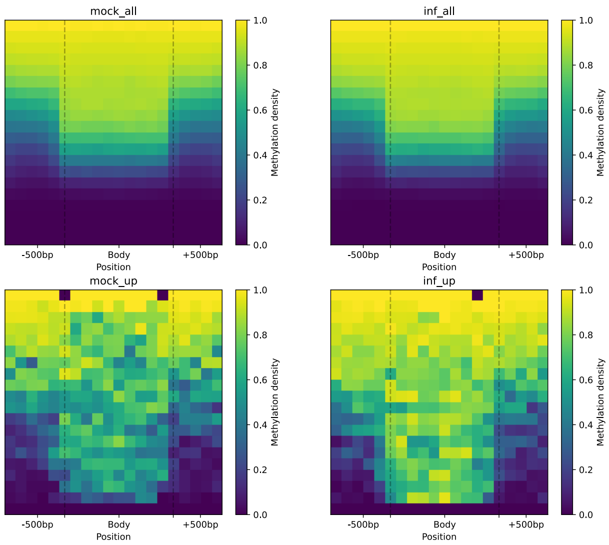

mock_all
inf_all
mock_up
inf_up
Methylation density
-500bp
Body
+500bp
Position

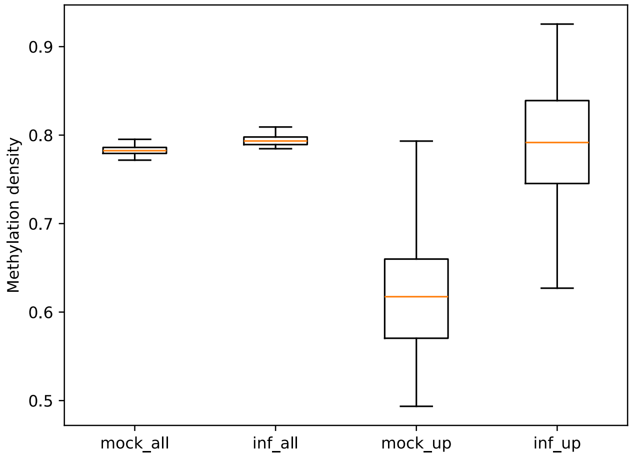

c. EDA of BS-seq data generated from in different non-model organisms (intra-species analysis in console mode)

BSXplorer enables comparison of methylation data across different organisms using the console command:

```
bsxplorer-metagene -o IntraMetageneReport --dir IntraMetagene -u 250 -d 250 -b
500 -S 50 --ticks \\-2000bp TSS Body TES \\+2000bp -C 0 -V 100 -H 100 --export
pdf intra_conf.tsv
```

A user can obtain a complete list of parameters by using the command ***bsxplorer-metagene --help*** The configuration file has the following structure

This header is for demonstration purposes only and it ***should not be*** *included in the conf.tsv.*					
Sample group	Path to report	Path to genome	Flank length	Minimal length	Region_type
Mus	SRR16815382_Mus_musculus.CX_report.gz	Mus_musculus_genomic.gff	2000	0	gene
Arabidopsis	A_thaliana.txt	A_thaliana_genomic.gff	2000	0	gene
Brachipodium	Brachypodium_distachyon_leaf.txt	Brachypodium_distachyon_genomic.gff	2000	0	gene
Cucumis	C_sativus.txt	C_sativus_genomic.gff	2000	0	gene
Brassica	DRR336466.CX_report.txt.gz	genomic.gff	2000	0	gene
Brassica	DRR336467.CX_report.txt.gz	genomic.gff	2000	0	gene

Below is a list of plots generated for the CG methylation context, as presented in the HTML report file.

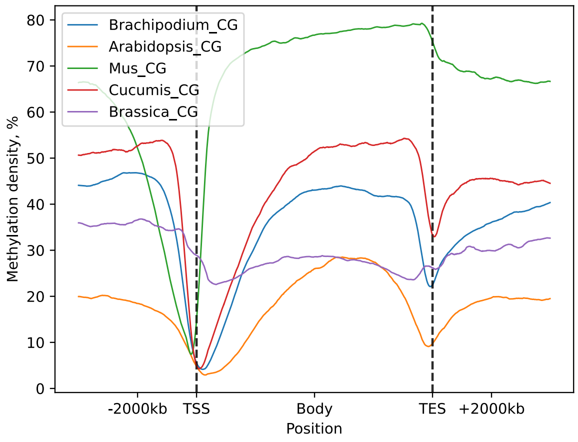

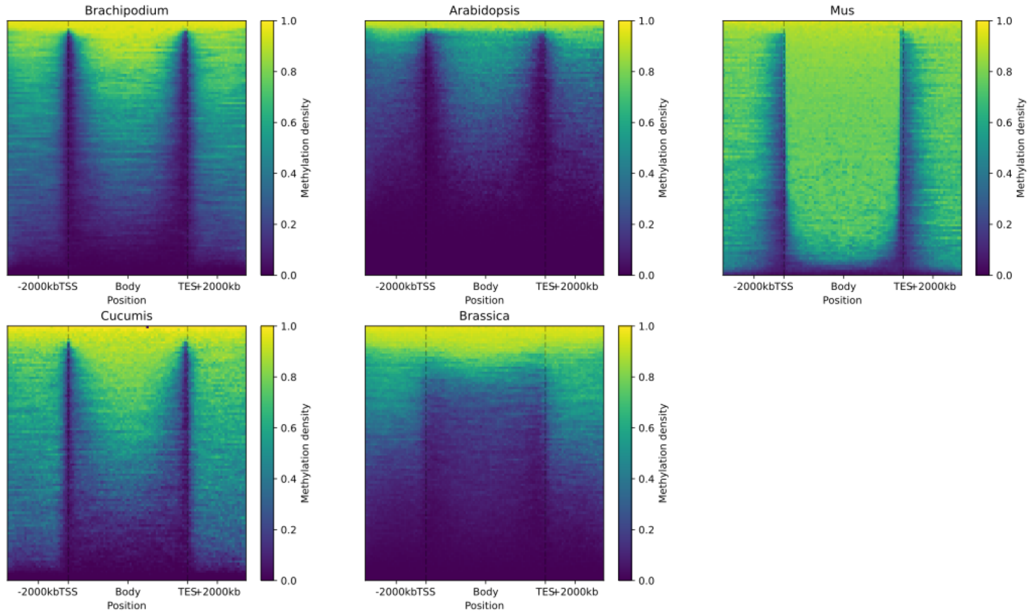

References:

1. Tirnaz S, Miyaji N, Takuno S, Bayer PE, Shimizu M, Akter MstA, et al. Whole-Genome DNA Methylation Analysis in Brassica rapa subsp. perviridis in Response to Albugo candida Infection. Front Plant Sci. 2022;13:849358.

2. Takuno S, Gaut BS. Body-Methylated Genes in Arabidopsis thaliana Are Functionally Important and Evolve Slowly. Mol Biol Evol. 2012;29:219–27.

3. Takuno S, Gaut BS. Gene body methylation is conserved between plant orthologs and is of evolutionary consequence. Proc Natl Acad Sci. 2013;110:1797–802.
